# Modelling the evolution of the European high-speed rail infrastructure network

**DOI:** 10.1038/s41598-025-34669-x

**Published:** 2026-03-06

**Authors:** Filippo Borgogno, Renzo Massobrio, Jorik Grolle, Oded Cats

**Affiliations:** 1 Future Cities and Communities Domain, LINKS Foundation, 10138 Torino, Italy; 2 Transport & Planning Department, Delft University of Technology, Delft, 2628CN The Netherlands; 3 Electronics & ICT Department, University of Antwerp, 2020 Antwerp, Belgium; 4 Mobility Transition Department, Haskoning, 3818EX Amersfoort, The Netherlands

**Keywords:** Energy and society, Energy science and technology, Engineering

## Abstract

High-speed rail (HSR) is often considered a promising and sustainable alternative for long-distance travel in the European context, aligned with Europe’s ambitious mobility and climate goals for 2050. However, a cohesive European HSR network is yet to be realised. Critically, the planning of a European HSR network requires considering how the network is to gradually evolve from its current fragmented state. We introduce an Evolutionary Network Growth model with Infrastructure and Network Effects considerations for European Rail (ENGINEER). This novel iterative network growth model selects the HSR infrastructure with the highest economic potential, continuously updating network configurations and demand patterns, subject to budget feasibility constraints. ENGINEER integrates cost estimates based on a microscopic representation and benefits estimated based on a macroscopic travel demand representation and is applied across 28 European countries. Our findings highlight the importance of path dependency and the benefits of an integrated decision-making in infrastructure planning. Model results demonstrate that ENGINEER can effectively identify promising HSR investments, yielding a cohesive and well-integrated European HSR network which leads to an increase in rail mode share per trip from 13% in 2023 to 27% by 2065.

## Introduction

In recent years, rail transport has gained significant attention in Europe as an attractive and sustainable mode of long-distance travel^1^. The European Union’s push for an integrated European railway network, supported by policy initiatives^2,3^, has revitalized rail’s importance. However, despite these efforts, cross-border rail connections and usage remain limited. Furthermore, given the current network state, a substantial investment—estimated at €25 billion per year—is required for the EU to meet its climate strategy, considering the critical role of modal shift in attaining related goals^4^.

High-speed rail (HSR) offers a promising solution, providing speed, accessibility, and sustainability advantages. Ambitious goals have been set to develop a comprehensive HSR network that aligns with future expansion potential^2,3^. Notwithstanding, the realization of a European HSR network faces substantial challenges, particularly in coordinating efforts among participating countries, as national priorities often hinder cross-border alignment. An assessment of European HSR infrastructural projects^5^ has highlighted cost overruns, delays, and poor financial management during project execution, leading to a fragmented network of poorly connected sub-networks. Tensions persist between preserving national autonomy in investment decisions and achieving a seamlessly interconnected European HSR network^5^. Past research demonstrated how an evaluation of cross-border connections requires a trans-national perspective in order to adequately account for related network effects or otherwise is destined to result in a sub-optimal outcome^6^. A coordinated decision-making process for a European HSR infrastructure planning has the potential to offer substantial benefits and result in a more cohesive and efficiently connected network. Related efforts to investigate the prospects of a European HSR network, in particular in terms of economic returns have culminated in the planning of the so-called Trans-European Transport Network (TEN-T)^7,8^. A recently released EU Master Plan on High-Speed Rail ^9^ outlines high-level goals and projected travel-time improvements but does not provide new detailed infrastructure plans. For network-level analysis, the TEN-T framework remains the most concrete and widely used reference.

The aforementioned planning efforts and past related studies adopted a static perspective on rail network planning. The network design problem was hence formulated and solved as a single optimisation decision. This was done for the design of the Chinese HSR network using a profit-oriented objective^10^ as well as for the design of the European service and frequency using various objective functions, including variants which account for societal welfare accounting for system externalities^11^. These studies designed and assessed the implications of future HSR networks for a given target year (i.e. 2030 or 2050), however, without considering how the network is to gradually develop towards it from its current state. Moreover, these studies did not examine in any detail aspects pertaining to infrastructural costs, which can be determinant for economic feasibility.

To this end, we propose a novel iterative network growth model to develop a long-term infrastructure HSR investment plan. The proposed ’Evolutionary Network Growth model with Infrastructure and Network Effects considerations for European Rail’, ENGINEER, is an evolutionary network-growth framework that simulates the long-term development of a European high-speed rail network by jointly evaluating infrastructure costs, travel demand, and network effects. It integrates a detailed spatial representation of Europe through a 3D hexagonal grid for cost estimation together with a macroscopic layer of major urban hubs for modelling demand and potential rail corridors. Travel generation, distribution, mode choice, and assignment are captured using a standard four-step transport modelling approach, allowing the model to quantify the benefits of travel-time savings and modal shifts. Candidate HSR links are assessed through cost–benefit analysis, and the network grows iteratively by selecting economically justified investments each year under a budget constraint, updating demand, travel times, and network structure after each addition. ENGINEER incorporates existing HSR infrastructure data, as well as population density, terrain coverage, and Price Level Index (PLI), to estimate infrastructural costs, consider feasible future expansions and assess alternative developments.

**Fig. 1:**
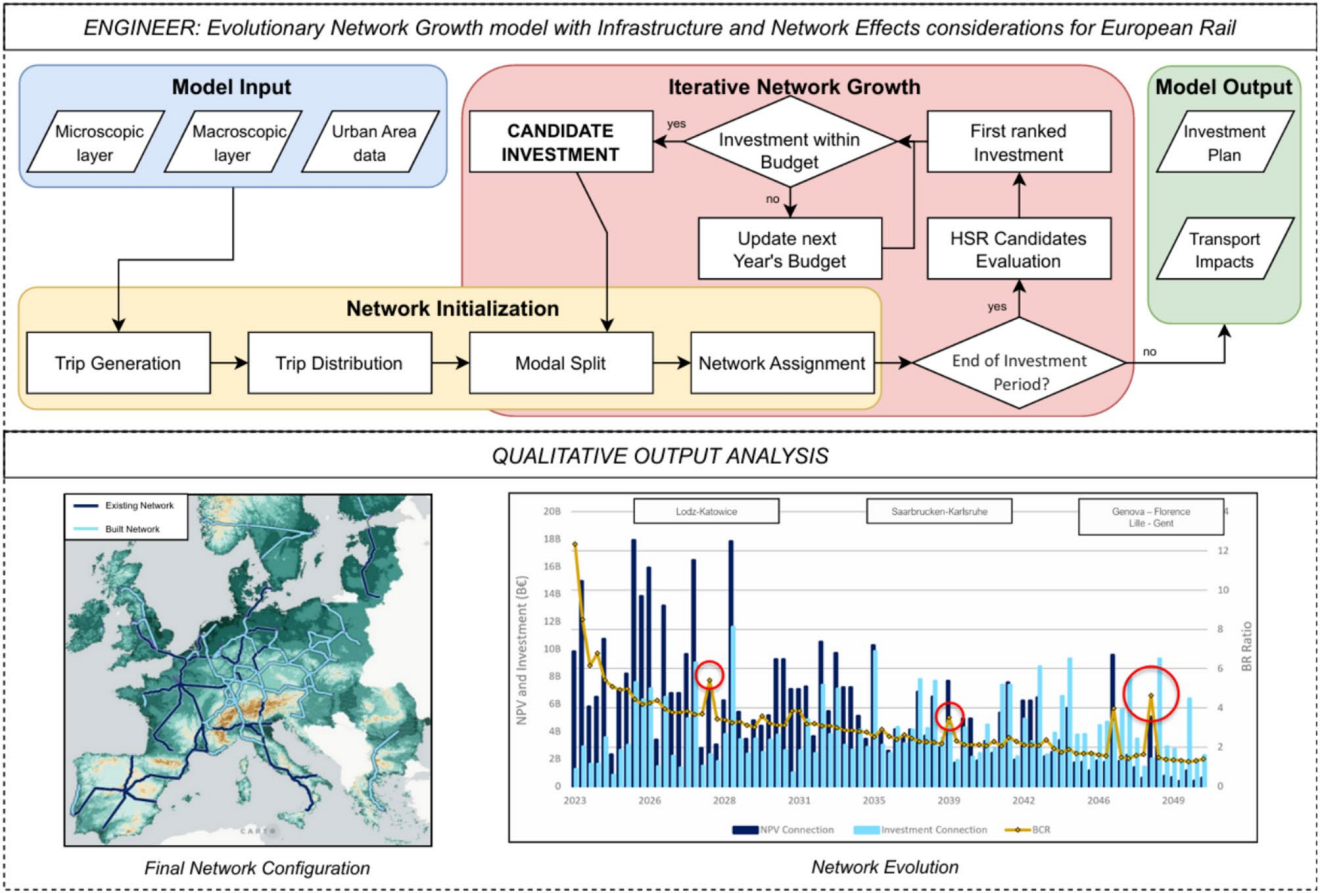
ENGINEER: an Evolutionary Network Growth model with Infrastructure and Network Effects considerations for European Rail. As shown at the upper panel, the model receives as input a multi-layer graph consisting of micro- and macro-scopic layers and the corresponding urban areas data. Following the estimation of travel demand between each origin-destination pair, ENGINEER applies an iterative network growth procedure which involves estimating modal split and the resulting HSR passenger flows for each candidate network state. Alternative investments are assessed by estimating the associated costs and benefits and are subject to annual budget constraints. The map at the bottom-left illustrates the resulting 2065 network configuration, with existing HSR in dark blue and newly built links in light blue. The plot at the bottom-right displays the evolution of investments, Net Present Value (NPV), and Benefit-Cost Ratio (BCR), with red circles marking BCR spikes driven by path dependency dynamics.

Although the problem could be framed as a large-scale network optimization problem, the iterative growth model provides greater transparency by reflecting real-world planning cycles. It enables step-by-step investment decisions, clarifying the evolving relationship between costs, demand, and accessibility. ENGINEER starts by defining potential HSR infrastructural connections by means of a detailed cost analysis, incorporating spatial constraints (microscopic layer) based on a detailed representation of topographical considerations (macroscopic layer). Long-distance transport demand between urban centers is then estimated, and assigned to different modes (i.e., car, rail, and air) and routes based on travel utilities for car, rail, and plane travel alternatives. Finally, the iterative process invests in the HSR infrastructure with the highest economic potential, continuously updating network configurations and demand patterns until the most efficient HSR network is established, subject to budget feasibility constraints. By capturing the dynamic interplay between infrastructure costs and demand distribution, the model determines where, when, and at what cost HSR infrastructure should be developed. The model is applied across 28 European countries, including EU members, Norway, Switzerland, and the UK, accounting for existing and planned HSR lines while aligning with the EU’s 2030 and 2050 investment milestones. Results demonstrate that the model effectively identifies optimal HSR investments, yielding a cohesive and well-integrated European HSR network. Figure 1 offers a schematic presentation of the adopted methodology, consisting of an algorithmic flow and a qualitative output analysis.

## Results

The results section presents the spatial evolution of the European HSR network, followed by an analysis of costs-benefits performance, shifts in modal share, and the broader benefits of a coordinated, integrated European investment strategy.

### A European HSR network

**Fig. 2:**
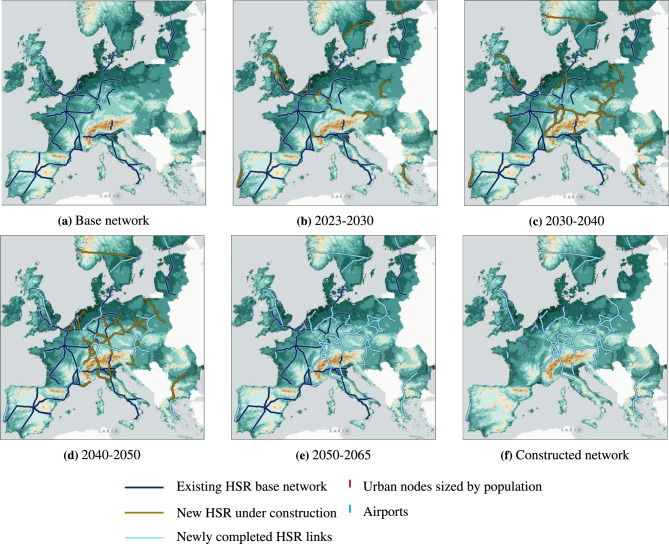
Dynamic network evolution 2023–2065. Links in dark blue represent the existing HSR base network, orange links correspond to investments under construction during the respective period, and those in light blue highlights completed investments which become operational within the same period. **(a)** Existing network. **(b)** 2023–2030 network expansion. **(c)** 2030–2040 network expansion. **(d)** 2043–2050 network expansion. **(e)** 2050–2065 network expansion. **(f)** Infrastructure built within the entire period (2023–2065).

Figure 2 presents the resulting network configuration across selected time periods, alongside the 2023 initial base network state mapping existing and planned HSR infrastructure (Fig. 2a). Based on historical budget trends, the initial network length of 10,951 km is expanded through iterative model-driven investments, adding 13,203 km of new connections, thereby bringing the total network length to 24,154 km. Notable growth is observed in central parts of Europe, most notably in Germany, Poland, Czech Republic and Austria, as well as in Switzerland and the United Kingdom, which are either situated between centres of high travel demand or itself being a high demand area, respectively. In contrast, countries like Spain, which already have extensive HSR networks, receive limited or no new investments. This indicates an iterative sequencing where high-demand, under-served regions are prioritized first, while saturated or peripheral countries follow later or are omitted. HSR network densification compared to 2023 occurs primarily in Switzerland, Germany, Belgium, Poland, and northern Italy along dense urban settlements, in some cases where HSR already exists (i.e. Northern Italy). Several new peripheral connections emerge, including Athens–Bucharest, Stockholm–Bergen, Stockholm–Gothenburg, and Lisbon–Porto, due to significant travel time savings. Denmark, Estonia, Latvia, and Lithuania see no network extensions. In the final scenario, rail market share increases from 13 to 27% between 2023 and 2065, increasing the competitiveness of high-speed rail to air over longer distances. This, on a network perspective, is translated into a shift of the break-even distance at which rail and air have an equal market share, from 380 km to 640 km. To achieve this growth, the model forecasts a total investment of €269 billion (2023 euros), progressively allocated between 2023 and 2050, resulting in a Net Present Value (NPV) of €563 billion. The final Benefit-Cost Ratio (BCR) of the investment is 3, indicating strong economic viability.

### Network evolution

We now take a closer look at the structural development stages of the resulting network. Figure 2 highlights three key iterative evolution phases generated by the model: early investments (Fig. 2b) focus on connecting major urban centers and closing gaps between (national) sub-networks, mid-phase expansions (Fig. 2c) extend connections over longer distances, while during the final densification phase (Fig. 2d), the network is strengthened around major hubs. This evolution reflects both preferential attachment and path dependency dynamics in network growth. In the initial early investments phase, expansion such as the Brussels-Antwerpen, Düsseldorf-Ruhr area and Mannheim-Frankfurt HSR lines, are heavily influenced by significant existing demand volumes and existing infrastructures, often closing gaps between existing networks. This follows the theory of preferential attachment^12^ which is driven by the “rich getting richer” principle. This pattern is especially pronounced at early stages of network’s growth, where regions with significant population density and existing infrastructure are favored by the model. As the network expands, investments in long-distance connections gain importance. Investments shift toward links which enable connecting regions across greater distances, exemplified by projects such as Stockholm–Gothenburg and Katowice–Kraków. These decisions are driven less by existing infrastructure and more by potential travel time reductions, demand growth and network effects, emphasizing the interplay between the value of time (VoT), distance, and population density in shaping network evolution through additional demand and competition between travel modes in the long-distance transport market. In the final densification phase, the model prioritizes strengthening the network by introducing alternative routes and travel shortcuts. Examples include the Genova–Florence and Ghent–Antwerp connections, which complement existing infrastructure to improve efficiency. At this stage, investments target marginal benefits, particularly in areas where further travel time optimization is possible, triggering a second wave of “rich get richer” effects. A rare exception is the Leeds–London shortcut, which was built as early as 2029 due to high demand flows and VoT, justifying early inclusion in the network.

**Fig. 3:**
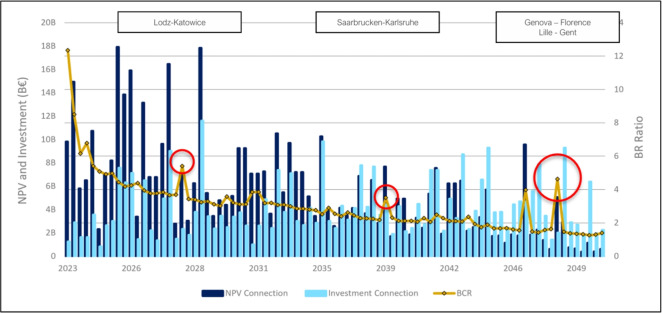
Iterative evolution of NPV, BCR and investment costs. For each investment, the NPV, investment costs, and BCR are presented. Red circles indicate instances where prior investments influence the BCR, reflecting network-wide interdependencies.

While the model exhibits preferential attachment tendencies, it also incorporates path dependency dynamics, where current investments are shaped by previously built infrastructure. This has also been observed in the context of metropolitan and regional network growth patterns^13,14^. Figure 3 shows the year-by-year evolution of infrastructural NPV, investment costs, and BCR, where NPV reflects total discounted benefits minus costs, and BCR expresses the ratio between them to assess economic efficiency. The decreasing trend line for of the BCR evolution reveals how these values are closely linked to prior investment decisions. The few notable exceptions to the otherwise decreasing trend in terms of BCR values correspond to sequential investments which are made beneficial as a consequence of the first decision in a sequence. This is observed for Warsaw–Łódź followed by Łódź–Katowice (2027), the Saarbrücken–Karlsruhe investment following the Luxembourg–Saarbrücken (2039) link construction, or Milan–Genoa followed by Genoa–Florence (2048), in all cases taking place in the subsequent year. All these connections become economically feasible as prior travel time reductions increase demand for subsequent expansions, demonstrating how past decisions shape future expansions. The last investment is scheduled for 2050, aligning with EU transport milestones^15^, while the final year 2065 reflects the completion of construction and full realization of benefits, accounting for an average 15-year planning and construction duration.

**Fig. 4:**
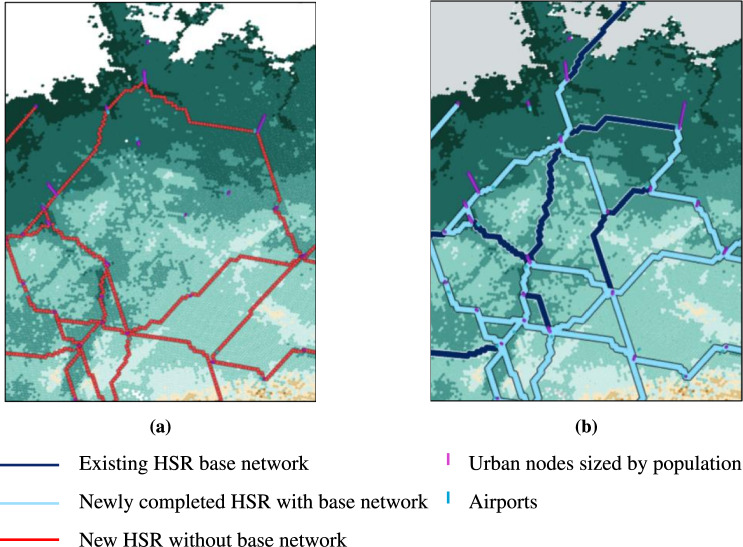
German HSR Network Evolution under different Base Network Scenarios. Network evolution in Germany from 2023 to 2065 with **(a)** an empty base network and **(b)** an initialized base network. Dark blue links represent the existing HSR base network, while light blue highlights newly added investments.

While no clear trend emerges regarding investment costs, later-stage projects tend to be more expensive and yield lower returns, reflecting how the model prioritizes high-benefit, cost-efficient investments earlier in the timeline. The role of preferential attachment and path dependency is further demonstrated in a scenario comparison where we contrast network expansions with and without taking the existing and planned infrastructure as given in the initial network state. Figure 4 shows the different network configurations for Germany under these two conditions. Without pre-existing infrastructure, the model optimizes return on investment by routing the Berlin–Munich connection via Dresden and Prague, where higher passenger volumes are expected. However, when existing infrastructure is considered, the model instead extends the connection along the existing Leipzig–Erfurt–Nuremberg corridor, where lower traffic volumes are compensated by reduced construction costs given past investments.

**Fig. 5:**
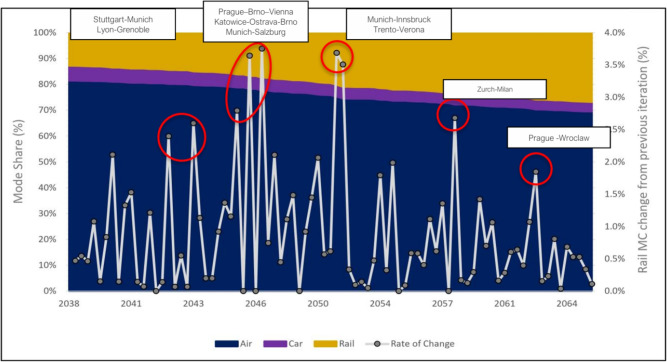
Mode share evolution over the investment period 2023-2065. The graph starts from 2038 since this is the year at which the first investment becomes operational. The grey line shows the year-on-year percentage change in rail mode share, while red circles highlight the most significant improvements.

The development of the rail network throughout the main analysis scenario results in an increase in rail mode share per trip from 13% in 2023 to 27% by 2065 (yellow area), as can be seen in Fig. 5. The relatively small share of car trips can be attributed to the fact that the demand data is dominated by long distances and is limited to demand between urban centers. This mode split refers only to inter-city long-distance trips and excludes user groups who typically do not consider public transport (e.g., lease car drivers). The trend line (in grey) shows the year-on-year percentage change in rail mode share compared to the previous iteration. Red circles mark years in which key investments have led to a significant growth in the market share of rail. Notably, these peaks align with major cross-border investments and trans-continental connections, emphasizing the critical role such infrastructure play in enhancing rail’s competitiveness. The first peak (2043) corresponds to the Stuttgart–Munich connection, a key east-west link that enables faster connections from Benelux and France to Austria, Hungary and Poland, which will undergo further densification at later stages. Additionally, the same year sees the opening of the Turin–Lyon cross-border corridor, by means of connecting Grenoble and Lyon, which plays a vital role in connecting the Italian HSR network with Western Europe’s HSR lines. The second peak (2046–2047) corresponds to the construction of multiple cross-border links in Central Europe between Poland, Austria, the Czech Republic, and Germany, demonstrating how path dependency iteratively strengthens passenger flows and improves the economic viability of subsequent investments. The third (2051) and fourth (2058) peaks coincide with Italy’s enhanced connectivity with Austria and Switzerland, including the Munich–Innsbruck, Trento–Verona, and Milan–Zurich corridors. The fifth and final peak (2063) marks another key international link connecting Poland (Wroclaw) to the Czech Republic (Prague) and onward to central Germany (Nuremberg). The dominance of cross-border investments in rail share spikes can be partially attributed to the fact that unlike international connections, most key national links already exist in the base scenario. Nevertheless, our findings underscore that mode share evolution patterns highlight significant untapped potential in international connections. Without a European-scale approach to network development analysis and investment appraisal, these projects would be difficult to justify within national frameworks, as their benefits extend beyond individual countries. Only coordinated decision-making at the European level can fully unlock their potential by capturing cross-border demand, reducing travel times, and thereby contributing to network-wide efficiency.

### The benefits of an integrated decision-making process

**Fig. 6:**
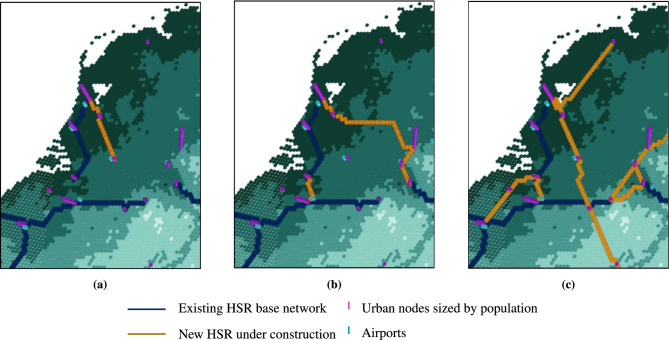
Duth HSR Network Evolution under different development perspectives. Network evolution from 2023 to 2065 under **(a)** national, **(b)** cross-border and **(c)** European perspectives. Dark blue links represent the existing HSR base network, while orange correspond to investments under construction that will become operational within the analysis period.

To illustrate the model’s decision-making process, we introduce a complementary experiment focusing on the Netherlands. Figure 6 compares investment outcomes under national, cross-border, and European planning perspectives. In the national scenario, Fig. 6a, constrained within Dutch borders, the model prioritizes internal connectivity, constructing the Amsterdam–Utrecht–Eindhoven corridor. This reflects a domestic-focused approach, optimizing national mobility while disregarding broader network efficiency. Taking a cross-border perspective with connections beyond the Netherlands, Fig. 6b, leads to the construction of Antwerp–Brussels and Amsterdam–Ruhr area via Utrecht. Notably, the Brussels–Antwerp link, located outside of the Netherlands, yields greater benefits to the Netherlands than an internal connection, while the Amsterdam–Ruhr area link delivers a higher return on investment than Amsterdam–Eindhoven, underscoring the added value of cross-border integration in boosting overall network efficiency. The European scenario, Fig. 6c, makes investment decisions at a continental scale, maintaining Brussels–Antwerp but selecting an alternative Amsterdam–Ruhr area route via Liège and Aachen. While less direct, this improves connectivity to European cities located further south such as Stuttgart, Zurich, and Milan, demonstrating how a European-wide appraisal perspective maximizes network-wide benefits by optimizing infrastructure investments to serve a larger demand pool while ensuring more cost-efficient allocation of resources.

**Fig. 7:**
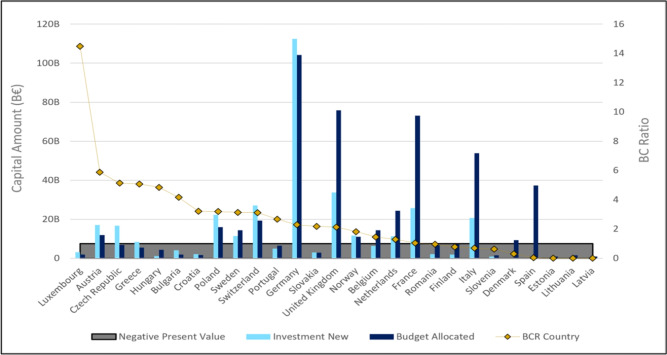
National economic indicators. Light blue lines indicate total investments per country, while dark blue lines show each country’s budget contribution. Yellow dots represent the BCR, with countries in the grey area facing a negative NPV, implying a financial loss.

Next, we analyze the country-specific consequences of the integrated planning of investments in a European HSR network. Figure 7 illustrates the distribution of investments, national contributions, and resulting economic returns. Importantly, the results highlight that even countries where no HSR investments are made within their borders, can still benefit from neighboring countries’ infrastructure developments, particularly through travel time reductions and externality savings. While these benefits are generally lower than those associated with local investments, accounting for cross-border spill-over effects in national appraisal processes may offer additional economical justification for certain projects, underscoring the importance of adopting a broader appraisal framework which considers effects extending beyond national borders.

Another key finding pertains to budget allocations under a centralized investment process (Fig. 7). Similar to the EU budget mechanism, where countries contribute to a common fund based on their wealth and the EU reallocates funds according to its priorities, the model shows that some countries contribute more than they directly benefit from the investment plan. The largest gap is observed for the United Kingdom, France, Italy, Spain and the Netherlands. While the United Kingdom still achieves a positive return on investment, others experience BCR values which are close to break-even or negative in case their higher contributions exceed their respective benefits. This suggests that, despite the benefits highlighted in the previous sections and a global BCR value of 3, an integrated decision-making process may result in some countries ending up with costs which exceed their benefits when taking a national perspective. These imbalances raise questions about how the distribution impacts of network developments could be addressed - an issue further explored in the discussion section.

### Comparison with TEN-T Milestone Networks

**Fig. 8:**
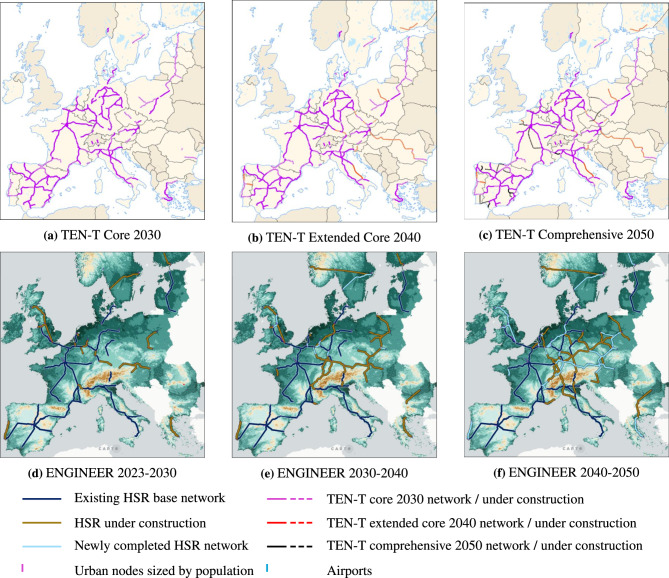
Comparison between ENGINEER and TEN-T Network evolutions. Links in the TEN-T plans represent HSR passenger connections with minimum speeds of more than 200 km/h, for 2030 (pink), 2040 (red) and 2050 (black). In the ENGINEER visualizations, links in dark blue represent the existing HSR base network, orange links correspond to investments under construction during the respective period, and those in light blue highlights completed investments which become operational within the same period. **(a)** TEN-T Core 2030 network. **(b)** TEN-T Extended Core 2040 network. **(c)** TEN-T Comprehensive 2050 network. **(d)** ENGINEER 2023-2030 network expansion. **(e)** ENGINEER 2030-2040 network expansion. **(f)** ENGINEER 2040-2050 network expansion..

To contextualize the evolution of the networks generated by ENGINEER and benchmark them against real-world planning trajectories, we compare our results with the staged HSR development outlined in the TEN-T framework. This framework defines a phased implementation strategy comprising the Core Network (2030), the Extended Core Network (2040), and the Comprehensive Network (2050) for HSR corridors with design speeds exceeding 200 km/h^16^. These layers represent the most widely adopted reference for European long-distance rail planning and therefore provide an appropriate baseline for comparison. It is important to note that ENGINEER identifies high-speed corridors at 250 km/h and above, whereas the TEN-T classification includes lines with design speeds exceeding 200 km/h. As a result, the TEN-T layers display a broader set of corridors than those considered within the model’s high-speed category. Figure 8 allows to visually contrast the evolution of the networks according to the TEN-T framework and as generated by ENGINEER.

At the 2030 horizon, the TEN-T Core network already forms a wide continental backbone, including major Iberian radials (e.g., Madrid–Lisbon, Barcelona–Valencia), cross-border sections such as Lyon–Turin and Bordeaux–San Sebastián, and eastward axes such as Warsaw–Kaunas, Warsaw–Wrocław/Poznań and Vienna–Linz. ENGINEER, which follows historical budget patterns, shows a more conservative expansion at this stage. The model concentrates on reinforcing the strongest early-demand corridors in Western and Central Europe, such as Brussels–Antwerp, the Rhine–Ruhr system and the Vienna–Bratislava–Budapest axis, while omitting high-cost projects through the Alps (e.g., Switzerland or the Brenner corridor) or across long distances (e.g., Czech Republic, Romania, Bulgaria and Greece). ENGINEER also identifies several short, high-return segments that TEN-T does not designate as standalone HSR links, such as Mannheim-Frankfurt, Aachen-Koln and Katowice-Cracow. Interestingly, ENGINEER develops new HSR infrastructure also in Sweden and Finland, as well as in the United Kingdom, only marginally considered in the TEN-T Core layer. Overall, these contrasts reflect TEN-T’s assumption of an extensive early network completion by 2030, while ENGINEER produces a more gradual and budget-consistent expansion driven by economic feasibility along concentrated demand patterns.

By 2040, the TEN-T Extended Core network expands further with major additions such as Vienna–Bucharest, Milan–Ljubljana, the Italian Adriatic corridor, the south Finnish corridor and multiple smaller gap-closing links including Stuttgart–Munich–Innsbruck, Brno–Prague–Dresden and Hannover-Bielefeld. In contrast, ENGINEER’s evolution focuses on consolidating and interconnecting existing clusters, strengthening links between Central and Eastern Europe, with Switzerland and Luxembourg emerging as important connectors. The model extends the network towards Poland and the Czech Republic, completes the France-Switzerland-South of Germany axis, connects Oslo and Stockholm, and reinforces the Hamburg–Rhine–Ruhr region. At this stage, the model still omits many of the longer TEN-T extensions to the Baltics, Romania and Greece, as well as parts of the Adriatic coast as well as the full Dresden-Prague–Vienna connection. Nevertheless, ENGINEER completes the Paris–Strasbourg–Stuttgart–Munich–Vienna–Budapest corridor earlier than TEN-T, illustrating how preferential attachment favours central, highly-populated and well-connected hubs. Additional reinforcement links also appear around major core nodes, which TEN-T does not list as distinct high-speed lines, such as connections towards Bern, Karlsruhe and Aachen. Overall, the 2040 comparison shows TEN-T advancing a broad, cohesion-driven expansion which aims at enlarging network coverage, while ENGINEER follows a more demand-weighted and path-dependent progression that strengthens the European core before extending towards the periphery.

By 2050, the TEN-T Comprehensive network reaches full continental coverage, with continuous high-speed corridors spanning almost all EU territories, including complete Baltic and Scandinavian axes, extensive Balkan coverage and a dense southern European HSR system. In comparison, ENGINEER’s 2050 network is substantially expanded but remains more concentrated around its main hubs: it consolidates a strong Western and Central European backbone (e.g., the Rhine-Rhur system and the Benelux area), adds limited extensions towards Poland, Austria and northern Italy, and reinforces selected north–south and trans-Alpine connections, such as Amsterdam-Milan-Florence, Warsaw-Prague-Stuttgart, Berlin-Vienna and Hannover-Munich-Verona. Yet, the generated network does not include many low-demand or high-cost segments such as the full Baltic mainline, the Budapest-Bucharest corridor or further extensions between Warsaw and Berlin or in the Iberian Peninsula.

At the 2050 final comparison stage, it is evident that the TEN-T network includes certain corridors which reflect either strong national agendas (e.g., HSR extentsions in Spain and Italy) or how EU cohesion and integration or geo-strategic goals can override economic feasibility (e.g., Budapest–Bucharest or the Baltic mainline). Conversely, ENGINEER consistently prioritises core regions such as the Rhine–Ruhr, the Benelux or the core network connection Lyon-Prague (omitted by TEN-T plans), due to their high demand and centrality. Taken together, these contrasts highlight that TEN-T embodies a blend of national constraints and political objectives, whereas ENGINEER follows a system-wide logic centred on efficiency and connectivity, resulting in a more concentrated but economically coherent long-term network.

## Discussion

We present a network growth model for developing a long-term, sequential investment in a European HSR network. The approach builds on a centralized appraisal and coordinated investment framework to maximize efficiency and resource allocation. Furthermore, special focus is given to realistically estimate investment costs, incorporating topographic and spatial constraints to enhance the accuracy of infrastructure cost estimations. Our findings highlight a dynamic interaction between passenger demand, travel time improvements, and infrastructure costs, shaping network’s evolution through three phases: (1) expansion between major urban centers and existing networks, (2) enabling long-distance extensions, and (3) core network densification. Investment patterns reflect preferential attachment, yet prioritization is also shaped by travel time savings, distance, and population. The findings also reveal path dependency, where subsequent investment decisions are strongly dependent on previous iterations, particularly in cross-border integration.

Most importantly, the findings underscore the added value of a centrally coordinated European approach (notably including the UK and Switzerland) to planning and investment. As shown in “Network evolution”, the model initially prioritizes network consolidation, suggesting that the existing network is still far from functionally integrated. The later emergence of international corridors highlights the growing importance of a European planning strategy. This reflects one of the main issues identified by the European Court of Auditors^5^, which characterized Europe’s HSR network as a patchwork of poorly connected national systems rather than a unified transnational infrastructure, due to national interests and a lack of cross-border planning coordination. The proposed methodology addresses these shortcomings by introducing a centralized appraisal framework and an iterative, demand-driven planning model. By extending the planning horizon and aligning investment decisions across borders, it enables phased integration - network consolidation followed by network extensions - showing how cross-border connectivity can become both economically justifiable and strategically sequenced over time.

Comparing model outcomes with the TEN-T plans^2^ reveals substantial differences in expected network structure and scale. Under the assumed continuation of historical budget trajectories, ENGINEER produces a more contained HSR expansion (roughly 13,000km) focused on a demand-driven prioritisation of core corridors, while the TEN-T results in a much larger network shaped by broader cohesion and integration objectives and is 50%^7^ to 250%^8^ longer than the 2050 network yielded by ENGINEER in our analysis. In terms of the investment choices made and the sequencing thereof, TEN-T and ENGINEER are often in agreement, likely because these routes represent the most essential links from both demand-driven and policy-oriented perspectives. This alignment is also influenced by the fact that both models rely on existing infrastructure, thereby reflecting path-dependency in relation to past investments. However, key differences emerge. TEN-T network differs by omitting several economically viable HSR corridors identified by the model (e.g., Milan–Genoa–Florence, Liège–Luxembourg–Saarbrücken, Lyon-Bern-Zurich-Stuttgart, Athens-Sofia-Bucharest, Oslo-Stockholm, and Dresden-Nuremberg-Stuttgart) while including links excluded by the model due to low economic feasibility (e.g., Bordeaux–San Sebastián, Budapest-Bucharest, Bologna-Bari , Warsaw-Kaunas). These divergences likely reflect the influence of coverage and integration objectives, political considerations, national infrastructure strategies (e.g., mixed-traffic versus dedicated HSR), and country-specific planning horizons that shape TEN-T decisions, rather than a primary focus on cost-effectiveness at the continental scale which is the driving force of investment decisions made by ENGINEER. Additional differences include our model’s omission of intermodal hubs, mixed traffic operations of freight and passenger services, and military or strategic uses of rail infrastructure - all relevant factors in comprehensive transport planning.

In this context, the proposed methodology offers a valuable tool for policymakers, appraisers, and researchers to support strategic HSR investment planning in Europe at a critical moment. Importantly, our findings indicate that meeting EU ridership and network objectives^2,5^ will require significantly higher investments and faster infrastructure construction than historically observed since our 2050 network results in a doubling rather than the EU goal of tripling HSR ridership by 2050^3^. The iterative network growth approach allows for prioritizing segments based on evolving demand and cost-effectiveness, contrasting with the broader, often politically driven scope of static network designs. This approach offers a practical framework enabling planning to extend further into the future by incorporating flexibility and feedback, allowing priorities to adapt in response to changing economic conditions, social dynamics, political landscapes, fiscal constraints, and technological advancements. Notwithstanding, investments in infrastructure must be accompanied by the efficient and effective design of international line services, the underlying rail traffic and attractive mobility offerings^17^.

Despite its merits, the model might underestimate demand flows and broader economic benefits, particularly for cross-border sections, due to limitations in capturing latent and induced demand, positive externalities, and wider socio-economic spillovers associated with international connectivity. Future research may expand the appraisal process to include wider economic impacts such as those related to labor and property markets. The negative returns found for some countries may have negative implications for public and political acceptance in such an integrated investment plan due to geographical disparities. Future work may incorporate into the model additional constraints to ensure that all countries involved at least break-even also when taking a national perspective on the results of the integrated plan. Further, corridor optimization and alternative route modeling could enhance cross-border connectivity and investment efficiency, as well as extend the prism to consider also network vulnerability^18^. Additionally, a deeper examination of the dynamic interaction between demand, infrastructure expansion and service design (i.e. lines, frequencies, capacities, prices) could offer insights into their long-term inter-dependencies and increased HSR’s potential attractiveness. Such developments will ultimately contribute to a comprehensive and effective framework for HSR network planning across Europe.

## Method

An Evolutionary Network Growth model with Infrastructure and Network Effects considerations for European Rail, ENGINEER, has been developed for the purpose of this study, with its components and workflow schematically illustrated in Fig. 1 and described in the following sections. ENGINEER consists of three interconnected modules: (i) Graph Representation, (ii) Network Initialization, and (iii) Iterative Network Growth. The Graph Representation module refers to aspects relevant for determining both the costs and benefits associated with a potential investment in high-speed rail (HSR). On the cost estimation side, it uses a hexagonal layer grid (microscopic layer) to accurately represent Europe’s topographical features in three dimensions, enabling cost assessments for both surface and underground infrastructure across varying terrain conditions. For a reliable estimate of potential benefits, the macroscopic layer grid identifies potential rail connections and corridors, determined by the significance of cities based on factors such as population and economic output parameters. The Network Initialization module initializes the transport demand distribution and mode utilities, applying a 4-step transport model formulation similarly to growth models developed for metropolitan and polycentric contexts^13,14^. Finally, in the Iterative Network Growth module, the model ranks HSR investments based on their BCR, with the first choice added at each iteration to the existing infrastructure of the Base Network. The process repeats until the yearly budget is fully utilized, at which point the model advances to the next year receiving a new budget allocation. The cycle continues until the target year 2065 is reached. In the following we detail each of the three modules included in our model.

### Graph representation module

We model the geography and HSR infrastructure using two layers: (i) The macroscopic layer identifies potential benefits associated with HSR developments based on relations between demand flows, whereas (ii) the microscopic layer enables estimating related investment costs using a detailed three-dimensional hexagon representation.

#### Microscopic layer specifications

This layer discretizes the topography using Uber’s H3 Hexagonal Indexing system^19^. Hexagons are refined with country-specific data, excluding sea areas, and enriched with altitude values using QGIS and Copernicus DEM raster data^20^. To incorporate topographical variations, the model employs a 3D multilayer hexagonal representation, where each hexagon is assigned layers corresponding to its altitude, thereby accommodating underground expansions and realistic terrain modeling. Each hexagon’s centroid is vertically spaced by 250 meters from centroids in higher or lower altitudes and horizontally spaced with 6 km distance from neighboring centroids. Neighboring hexagons are then linked to establish feasible centroid connections, forming an interconnected grid. Centroids are linked both horizontally and vertically, with the 250-meter elevation difference calibrated against the horizontal spacing between hexagons to ensure realistic slopes that remain within the 2.5% gradient limit defined by international HSR standards^21^, enabling both surface and underground connections. The next step involves assigning distinct cost values for surface and underground nodes reflecting the economic implications of constructing HSR infrastructure. These costs are primarily influenced by terrain, population density, and design choices^22–24^. Mountainous areas and densely populated areas are associated with higher costs, while gradients and terrain features add complexity. Sea-crossing infrastructure (bridges/tunnels) is manually added by selecting the sequence of hexagons with the associated costs based on past project documentation. Next, we estimate per-kilometer construction costs across Europe using the following procedure. First, an average construction costs per kilometer is calculated across the case study area for both surface and underground HSR infrastructure, based on data from past European HSR construction^25^. Second, a cost weight is calculated for each country, based on four parameters: share of rough national terrain, national population density per kilometer, national GDP, and national Price Level Index. Third, for each country, country-specific surface and underground infrastructure costs parameters are specified. These values are imputed per centroid and edge weights are then computed as the average cost of connected centroids multiplied by the respective distance. In this way, the microscopic layer incorporates the full cost of constructing the infrastructure, both above and below ground, so that traversing it directly reflects actual construction expenses. These costs are calculated as a building block for later use outside the iterative Network Growth module outlined in Fig. 1.

#### Macroscopic layer specifications

This layer models demographic and the resulting travel demand distribution by mapping major urban hubs connected by potential rail links, represented as nodes connected by edges. Nodes are geographically indexed and linked to the nearest microscopic grid hexagon, and are selected based on population and local GDP. The parameters associated with each node are urban area population, GDP per capita, altitude, population density and coordinates, along with language, country, Schengen area presence and country population. Once nodes are defined, edges representing rail connections between these hubs are initialized. Unlike previous studies that primarily reference TEN-T policies^7,8,11^, this work introduces a novel rail link selection methodology without relying on predefined schemes, allowing links to emerge based on their return for investment potential rather than political or historical priorities, thus supporting a more demand-driven network configuration. Following some experimentation, we found a distance of 130 km between sequentially connected nodes in the macroscopic layer to best balance between network connectivity and computational efficiency.

#### HSR infrastructure construction

We identify candidate HSR infrastructure investments by undertaking the following three steps: (1) identify a rail link in the macroscopic grid; (2) apply Dijkstra’s algorithm^26^ between the two heaxagons identifying the extremes of the link to find the most cost-effective hexagonal path in the microscopic grid; (3) calculate infrastructure costs as the sum-product of edge weights and the respective edge lengths as the number of hexagons times the distance between centroids. The infrastructure cost field on the microscopic hexagonal grid is computed once at initialization, while candidate links are evaluated iteratively during network growth. The method balances costs and travel times, allowing for higher-cost solutions (e.g., tunnels) when justified by travel time savings. Subsequently, direct city connections from the macroscopic grid are expanded into routes using the *k* shortest paths within time limits *t*. The set of alternative paths constitutes a tailored candidate investment pool for each Origin-Destination pair.

### Network initialization module

#### 4-Step transport demand modelling

We apply the 4-step transport modelling framework to the European long-distance passenger market by sequentially modelling trip generation and attraction, trip distribution, modal split and network assignment. In doing so, we adopted the approach taken in^11^ for estimating demand for long-distance travel in Europe. In the trip generation stage, annual trips per city are estimated by distinguishing between long- and short-distance travel, using Functional Urban Areas (FUAs) to capture commuting zones, and assuming that attraction equals production. Trip volumes are computed by multiplying population by the European average annual trips and adjusting by the ratio of city-level to average GDP per capita, capturing income effects on demand. In the trip distribution stage, trips are allocated across metropolitan areas using a gravity formulation, which includes friction factors (e.g., language, Schengen borders). We apply a doubly constrained gravity model, ensuring full trip distribution through iterative balancing of the origin-destination matrix. For mode choice, the Random Regret Minimization (RRM) model^27^ is applied for choosing an alternative compared to the performance of other alternatives as elaborated next. Finally, trips are assigned to the network using an All-or-Nothing principle.

#### Mode choice specifications

Mode choice between car, plane, conventional rail and HSR is determined based on the disutility associated with each travel alternative $m \in \{\text {car}, \text {plane}, \text {rail}, \text {HSR}\}$. Disutilitites are defined by the associated travel time components: access ($t_{\text {acc},m}$) , waiting ($t_{\text {accwait},m}$), in-vehicle ($t_{\text {trav},m}$), exit waiting ($t_{\text {egrwait},m}$) and egress times ($t_{\text {egr},m}$), and their respective coefficients, see Eq. 1. Table 1 details the parameter values and how travel time components are calculated for each of the modes considered.

1 $$\begin{aligned} U_m = \beta _{\text {acc}} \cdot t_{\text {acc},m} + \beta _{\text {accwait}} \cdot t_{\text {accwait},m} + \beta _{\text {trav}} \cdot t_{\text {trav},m} + \beta _{\text {egrwait}} \cdot t_{\text {egrwait},m} + \beta _{\text {egr}} \cdot T_{\text {egr},m} \end{aligned}$$

**Table 1:** Mode alternative utility function parameters.

	Access	Waiting	In-vehicle	Exit waiting	Egress
β coefficient^a^	$\beta _{\text {acc}} = 1.36$	$\beta _{\text {accwait}} = 1.5$	$\beta _{\text {trav}} = 1$	$\beta _{\text {egrwait}} = 1.5$	$\beta _{\text {egr}} = 1.36$
Car	-	$t_{\text {accwait},car} = 10\%$ $t_{\text {trav},car}^\textrm{j}$	$t_{\text {trav},car} =$ $\text {ORS}* 1.2^\textrm{b}$	-	-
Plane	$t_{\text {acc},plane} =$ $\text {ORS} * 1.61^\textrm{c}$	$t_{\text {accwait},plane} = 2h^\textrm{d}$	*t*_trav,plane_=	$t_{\text {egrwait},plane} =$ $0.5h^\textrm{d}$	$t_{\text {egr},plane} =$
			$\frac{GCD}{700km/h}^\textrm{e}$		$\text {ORS} * 1.61^\textrm{c}$
Conventional rail	$t_{\text {acc},rail} =$	$t_{\text {accwait},rail} = 0.25h^\textrm{f}$	*t*_trav,rail_=	- $^\textrm{f}$	$t_{\text {egr},rail} =$
	$\frac{\frac{1}{4} \text {City radius}}{30 \text { km/h}} * 1.1^\textrm{g}$		$\frac{ORS}{110km/h}*1.15^\textrm{h}$		$\frac{\frac{1}{4} \text { City radius}}{30 \text { km/h}}* 1.1^\textrm{g}$
HSR	$t_{\text {acc},HSR} =$	$t_{\text {accwait},HSR} = 0.25h^\textrm{f}$	*t*_trav,HSR_=	- $^\textrm{f}$	$t_{\text {egr},HSR} =$
	$\frac{\frac{1}{4} \text { City radius}}{30 \text { km/h}}* 1.1^\textrm{g}$		$\frac{MICRO layer}{220km/h}*1.09^\textrm{i}$		$\frac{\frac{1}{4}\text {City radius}}{30 \text { km/h}}* 1.1^\textrm{g}$

$^\textrm{a}$ The weights for the utility parameters are based on the values used by^11^.

$$^\textrm{b}$$ Calculated via

$^\textrm{c}$ OpenRouteService (ORS) between city coordinates, adjusted with 1.2 detour factor^28^. OpenRouteService’s API calculates distance between city and nearest airport^29^, adjusted by detour factor of 1.61^28^, same for egress.

$^\textrm{d}$ Security checks and on-boarding procedures during access, baggage collection and terminal exiting for egress^30^.

$^\textrm{e}$ Great Circle Distance divided using an average speed of 700 km/h^28^.

$^\textrm{f}$ Accounting for an arbitrary 15 minutes access time to locate the platform and reaching the carriage. Exit waiting time is considered negligible.

$^\textrm{g}$ Access and egress times are based on one-quarter of the city radius, divided by 30 km/h and adjusted by a 1.1 detour factor^28^, using city-specific data from the OECD FUA database^31^.

$^\textrm{h}$ Same distance assumed for car and calculated with OpenRouteService (ORS), divided by 110 km/h and adjusted by 1.15 detour factor^28^.

$^\textrm{i}$ Microscopic layer based weighted shortest path (as explained in Section 4.1) divided by 220 km/h and adjusted with a 1.2 detour factor^28^.

$^{\textrm{j}}$ Arbitrary penalty of 10% added to the total in-vehicle time of car, to simulate pauses and stops along the trip

### Iterative network growth module

We assess all candidate links and corridors based on their ability to attract demand via travel time improvements in relation to the associated costs. Benefits are compared against infrastructure costs by means of a Cost-Benefit Analysis to determine economic feasibility. To assess the potential benefits, we analyze demand shifts (e.g. increase in overall demand or changes in demand distribution) and mode choice, thereby allowing for quantifying time savings and externality reductions. At each iteration, the top-scoring candidate is selected, and the latest network state and travel data are updated. The Price Level Index ($PLI_c$) is used to adjust reference national VoT to account for purchasing power disparities, aligning investment appraisals with cost estimates. Externality savings, including reductions in air pollution, accidents, and noise, are considered as additional benefits in HSR appraisal. Based on CE Delft^32^, these are expressed in euro cents per passenger-kilometre. We evaluate the financial feasibility of alternative investments by calculating and comparing their NPV and BCR by contrasting infrastructure and maintenance costs with projected benefits via cash flow analysis. A 50-year investment horizon is used, including a fixed planning and construction phase of 15 years, together with a discount rate of 4.5% to determine the present value of future benefits and costs. The last investment is scheduled for 2050, aligning with EU transport milestones^15^.

#### Investment decision & network update

Finally, NPV and BCR are jointly used for project ranking: NPV ranks alternatives for any given OD pair, while BCR ranks projects across the entire experiment. The highest-scoring investment is selected based on BCR score and budget constraints. The annual budget is calculated as a GDP-based contribution from each country, in line with historical European HSR spending trends. Once selected, investments are integrated into the network, updating the network state, travel utilities, mode choices, and removing the selected link and corresponding OD pairs from the candidate pool. Importantly, new links can only be used by travelers once their construction has been completed. The built matrix is updated, travel times and mode shares recalculated, and the budget adjusted. If the budget is insufficient, the model advances to the next year, while any remaining funds are carried over. Finally, if no project yields a positive BCR, the iterative network growth process terminates.

## Data Availability

The datasets and code generated during and/or analyzed during the current study are available in the following repository https://doi.org/10.4121/314b4240-163d-406e-8498-716802c3627a
